# Synthesis and crystal structure of allyl 7-(di­ethyl­amino)-2-oxo-2*H*-chromene-3-carboxyl­ate

**DOI:** 10.1107/S2056989021002218

**Published:** 2021-03-02

**Authors:** Vanessa Nowatschin, Christian Näther, Ulrich Lüning

**Affiliations:** a Otto-Diels-Institut für Organische Chemie, Christian-Albrechts-Universität zu Kiel, Otto-Hahn-Platz 4, D-24098 Kiel, Germany; b Institut für Anorganische Chemie, Christian-Albrechts-Universität zu Kiel, Max-Eyth Str. 2, D-24118 Kiel, Germany

**Keywords:** crystal structure, synthesis, 2-oxo-2*H*-chromene, C—H⋯O hydrogen bonding

## Abstract

The title compound, C_17_H_19_NO_4_, was synthesized by the reaction of 7-(di­ethyl­amino)-2-oxo-2*H*-chromene-3-carb­oxy­lic acid with allyl bromide and purified by flash column chromatography on silica gel. Crystals suitable for single-crystal X-ray diffraction were obtained by recrystallization from acetone. The aromatic core of the mol­ecule is not planar with the di­ethyl­amino group and with the carboxyl group that are rotated out of the 2-oxo-2*H*-chromene plane by 6.7 (2)° and 11.4 (2)°. The NC_2_ unit of the di­ethyl­amino group is planar with an angle sum close to 360°. Inter­molecular C_ar_—H⋯O_carbon­yl_ inter­actions lead to the formation of chains parallel to the *b* axis. X-ray powder diffraction analysis proves that the title compound was obtained as a pure phase.

## Abstract 2

The crystal structure of the title compound, C_17_H_19_NO_4_, consists of nearly planar mol­ecules that are linked by inter­molecular C—H⋯O hydrogen bonding into chains along the *b-*axis direction.

## Chemical context   

Coumarins or 2*H*-1-benzo­pyran-2-ones are fluoro­phores with a wide range of biological and chemical applications (Bardajee *et al.*, 2006*a* ▸). One of the most important aspects is the detection of enzymatic activity from bacteria like *Enterococci* or *Streptococci* (Devriese *et al.*, 1999 ▸). Within the enzymatic reaction, naturally occurring aesculin is hydrolysed with a concomitant loss of fluorescence (Edberg *et al.*, 1976 ▸). In addition, (coumarin-4-yl)methyl esters are often used as a photocleavable protecting group that could be useful for proton detection in biological processes (Geissler *et al.*, 2005 ▸). Another emerging field of application is photoelectricity such as in organic light-emitting diodes (OLEDs) or laser dyes (Bardajee *et al.*, 2006**a* ▸;* Jones *et al.*, 1985 ▸; Jones & Rahman, 1992 ▸, 1994 ▸; Cui *et al.*, 2018 ▸). In this context, Cui *et al.* (2018 ▸) developed two coumarines that show solid-state fluorescence influenced by NH_3_ or HCl gas.

**Figure 1:**
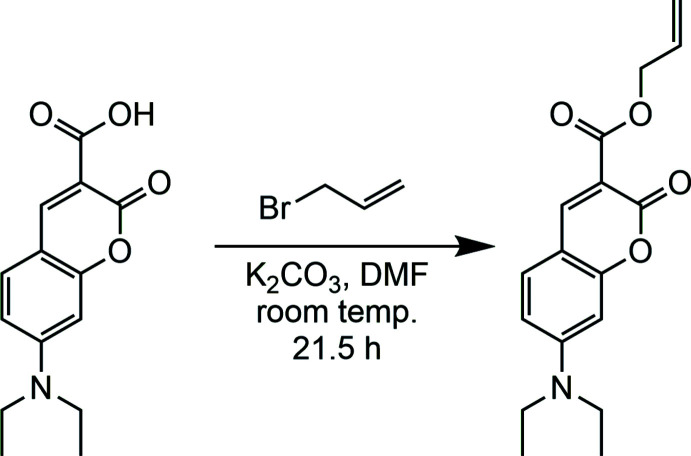
Synthesis of allyl 7-(di­ethyl­amino)-2-oxo-2*H*-chromene-3-carboxyl­ate by esterification of 7-(di­ethyl­amino)-2-oxo-2*H*-chromene-3-carb­oxy­lic acid with allyl bromide.

In a current research project, we planed to insert a coumarin moiety as part of a pH-sensitive polymer to visualize material damage. For this purpose, allyl 7-(di­ethyl­amino)-2-oxo-2*H*-chromene-3-carboxyl­ate was synthesized from 7-(di­ethyl­amino)-2-oxo-2*H*-chromene-3-carb­oxy­lic acid and allyl bromide with potassium carbonate for deprotonation and dry *N*,*N*-di­methyl­formamide as solvent (Fig. 1 ▸). The obtained title compound was characterized by ^1^H NMR (Fig. S1 in the supporting information) and ^13^C NMR (Fig. S2) spectroscopy, mass spectrometry, IR spectroscopy and elemental analysis. Recrystallization from acetone led to crystals that were characterized by single-crystal X-ray diffraction. Based on the results of the structure determination, a powder X-ray pattern was calculated and compared with the experimental pattern, revealing that the title compound was obtained as a pure phase (Fig. S3).
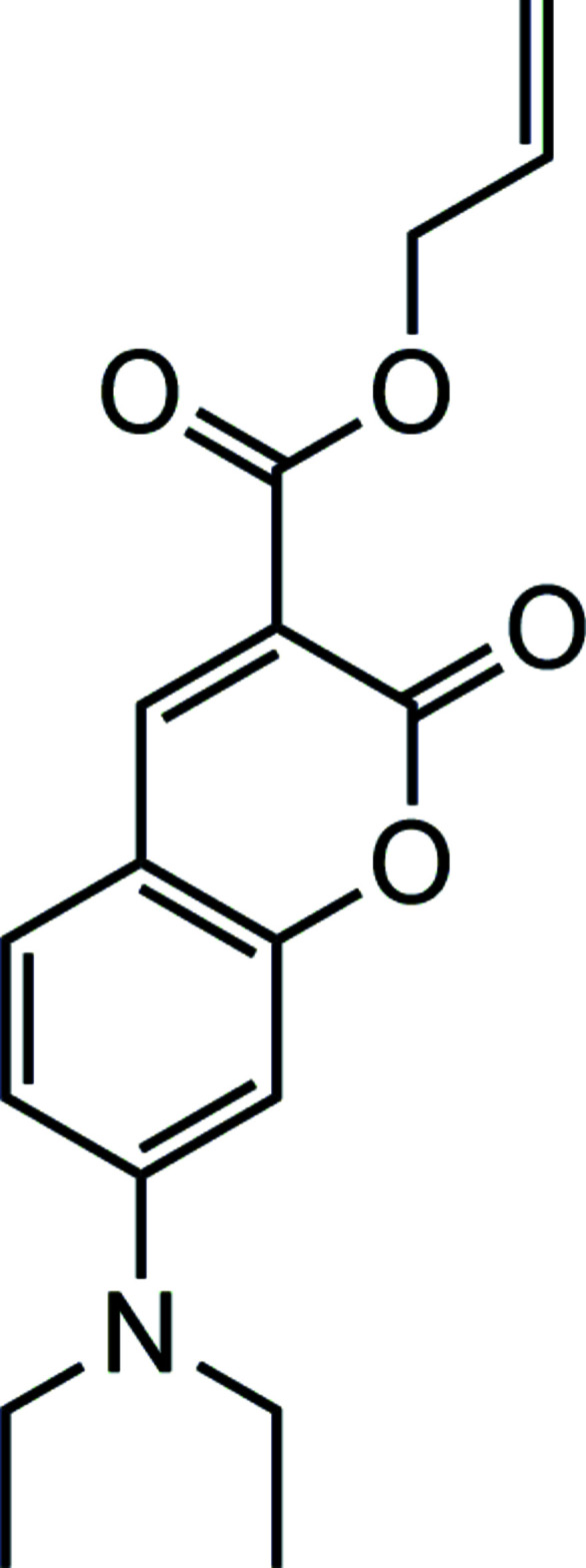


## Structural commentary   

**Figure 2:**
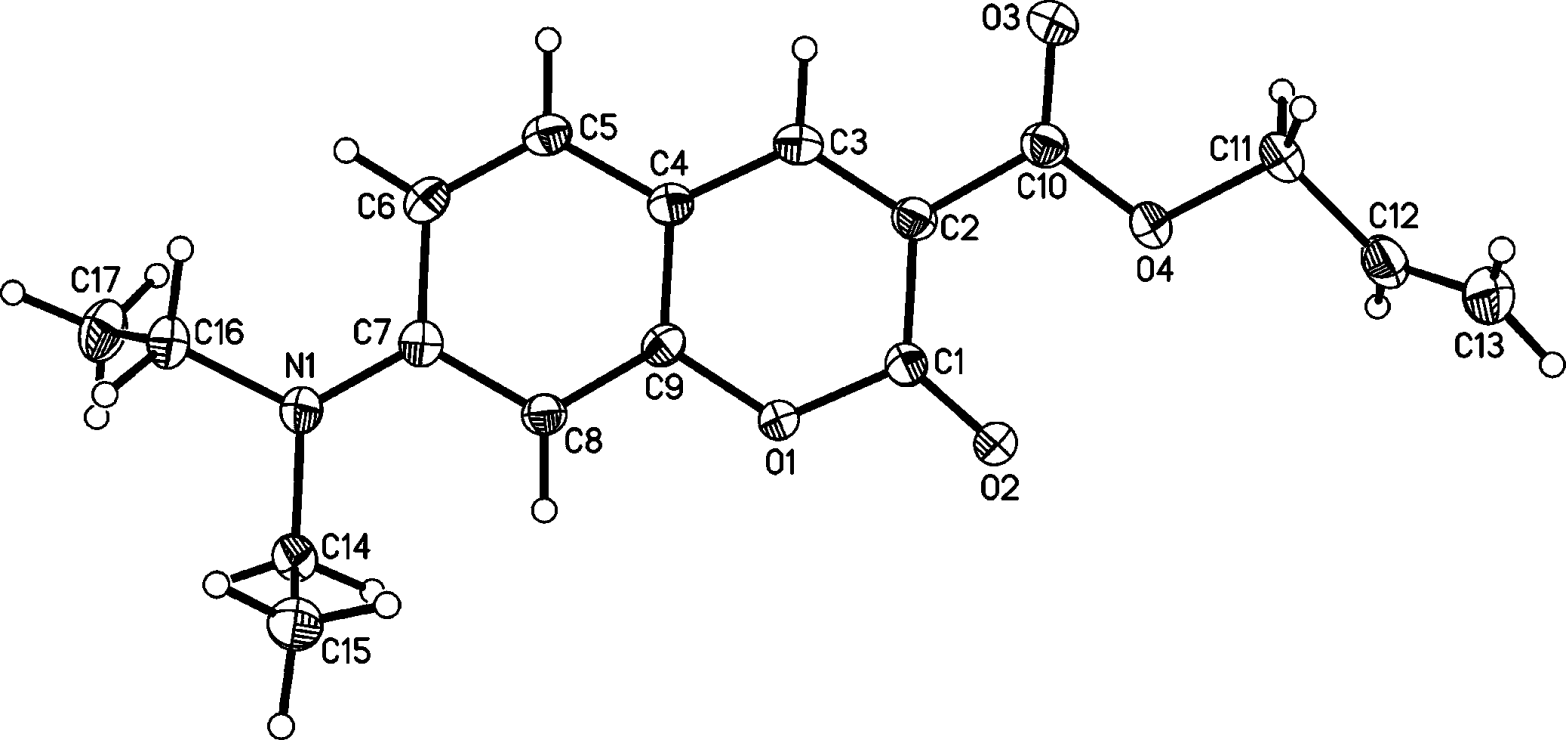
Mol­ecular structure of the title compound with atom labelling and displacement ellipsoids drawn at the 50% probability level.

**Figure 3:**
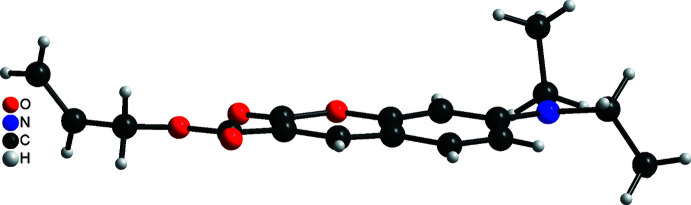
The orientation of the substituents in the mol­ecular structure of the title compound.

The mol­ecular structure of the title compound, C_17_H_19_NO_4_, consists of a central 2-oxo-2*H*-chromene (2-benzo­pyrane) unit with a carb­oxy­lic acid allyl ester in 3-position and a di­ethyl­amino group in 7-position. All atoms of the mol­ecule are in general positions (Fig. 2 ▸). The 2*H*-chromene unit is essentially planar with a maximum deviation for O2 of 0.1021 (6) Å from the least-squares plane calculated through C1–C7 and O1 and O2. The carboxyl group (C10,O3,O4) is slightly twisted from the 2-oxo-2*H*-chromene unit, with the dihedral angle between the plane calculated through the ring system and that of the carboxyl group being 6.7 (2)° (Fig. 3 ▸). The NC_3_ unit (N1,C7,C14,C16) of the di­ethyl­amino group is nearly planar with a maximum deviation of the N atom from the mean plane of 0.0873 Å; planarity is also obvious from the sum of the C—N—C angles of 358.9°. This unit is rotated from the 2-oxo-2*H*-chromene plane by 11.4 (2)° (Fig. 3 ▸), which points to conjugation between the ring system and the di­ethyl­amino group. The latter feature is also reflected by the C7—N1 bond length of 1.3597 (12)°.

## Supra­molecular features   

**Figure 4:**
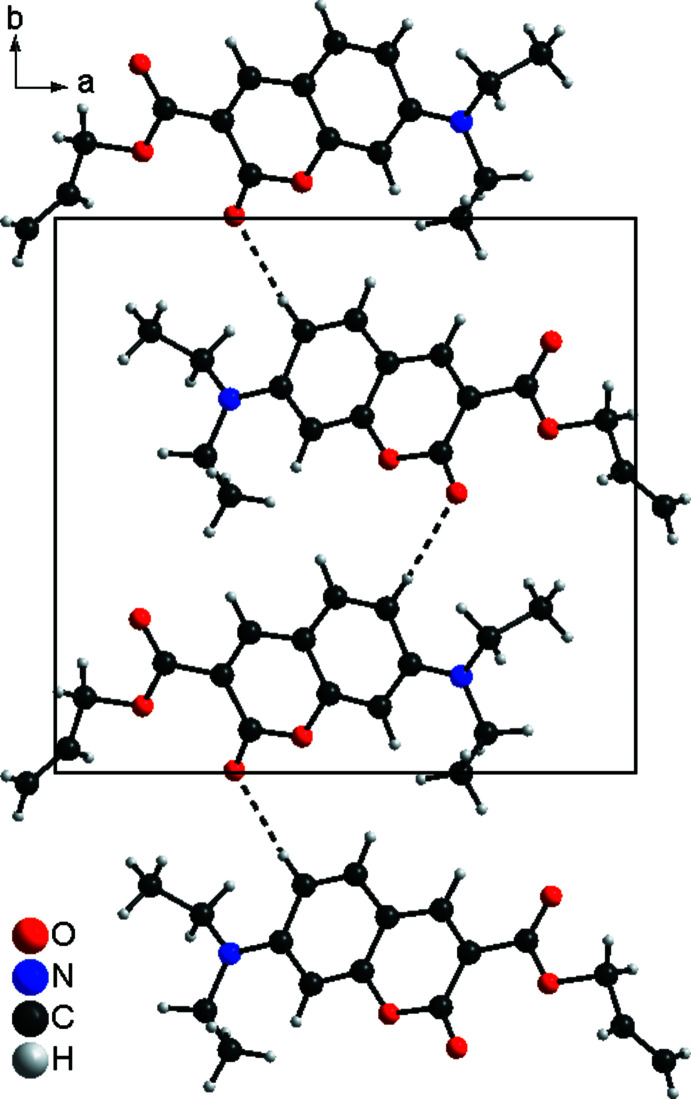
The formation of C—H⋯O hydrogen-bonded chains in the title compound in a view along the crystallographic *c* axis. Hydrogen bonds are shown as dashed lines.

**Figure 5:**
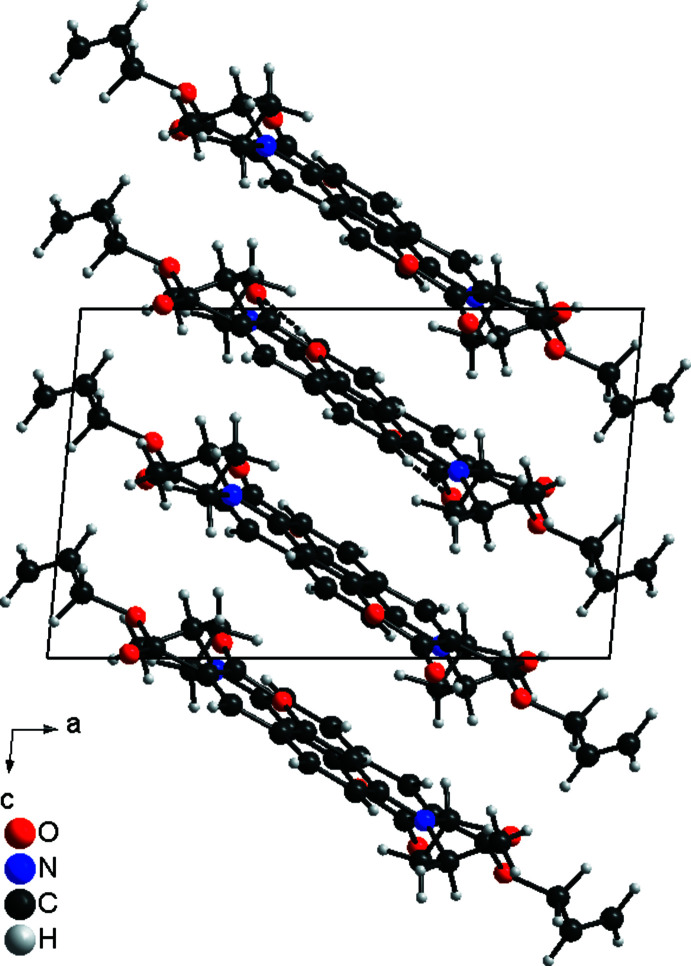
Packing of mol­ecules in the crystal structure of the title compound in a view along the crystallographic *b* axis. Inter­molecular C—H⋯O hydrogen bonding is shown as dashed lines.

In the crystal structure of the title compound, the mol­ecules are linked by inter­molecular C—H⋯O hydrogen bonding between one of the aromatic hydrogen atoms of a 2-oxo-2*H*-chromene unit and a carbonyl oxygen atom of a neighbouring mol­ecule into chains extending parallel to the crystallographic *b* axis (Fig. 4 ▸; Table 1 ▸). The C—H⋯O angle is close to linearity, indicating that this is a relatively strong inter­action. The mol­ecules are additionally stacked into columns that are directed along the crystallographic *c* axis but the mean planes of the 2*H*-chromene rings of neighbouring mol­ecules are not parallel (Fig. 5 ▸). They are rotated by 33.2°, which prevents π–π inter­actions.

## Database survey   

A search in the Cambridge Structural Database (CSD Version 2021; Groom *et al.*, 2016 ▸) revealed eight structures of 7-(di­ethyl­amino)-2-oxo-2*H*-chromene-3-carboxyl­ate derivatives. Three of them relate to the crystal structures of the carb­oxy­lic acid, which crystallizes in two different polymorphs (Bardajee *et al.*, 2006*a* ▸; Cui *et al.*, 2018 ▸; Zhang *et al.*, 2008 ▸).

Five more crystal structures relate to esterificated coumarin derivatives. One of them is 3-carb­oxy­ethyl-7-di­ethyl­amino­coumarin (Li *et al.*, 2009 ▸). Another one is succinimidyl 7-(di­ethyl­amino)-2-oxo-2*H*-chromene-3-carboxyl­ate, which was obtained as a chloro­form solvate (Bardajee *et al.*, 2006*b* ▸). The hits also include 4-cyano­biphenyl-4-yl 7-di­ethyl­amino-2-oxo-2*H*-chromene-3-carboxyl­ate (Sreenivasa *et al.*, 2013 ▸). Furthermore, two bis­chromophoric acid derivatives are reported. The first one is (2*R*,3*R*)-diethyl tartrate-2,3-bis­(7-di­ethyl­amino­coumarin-3-carboxyl­ate) and the second is (2*S*,3*R*)-*N*,*O*-bis­(7-di­ethyl­amino­coumarin-3-carbon­yl)-threonine methyl ester (Lo *et al.*, 2001 ▸).

## Synthesis and crystallization   

All reagents and solvents were commercially available and were used without further purification: allyl bromide (abcr), 7-(di­ethyl­amino)-2-oxo-2*H*-chromene-3-carb­oxy­lic acid (Fluoro­chem). For the reaction, flasks were flame-dried, evacuated and flooded with a stream of nitro­gen. The NMR spectra were measured with a Bruker AvanceNeo 500 (^1^H NMR: 500 MHz, ^13^C NMR: 125 MHz) in di­methyl­sulfoxide-*d*_6_ (deutero) as solvent. TMS was used as reference. The melting point was measured with a Melting Point Apparatus from Electrothermal. The mass spectrum was measured in the positive mode with an AccuTOF GCV 4G (Jeol, EI, 70 eV). *R*_f_ values were determined by thin-layer chromatography using ALUGRAMM^®^ Xtra Sil G/UV_254_ plates (Machery-Nagel). Flash column chromatography was performed using cartridge SNAP Ultra 25 g (Biotage^®^) on a Isolera one (Biotage^®^). Infrared spectroscopy was performed on a Perkin–Elmer 1600 series FTIR spectrometer. An AG531-G Golden-Gate-Diamond-ATR unit was used. The elemental analysis was performed with a vario MICRO CUBE (Elementar). The probe was put into a zinc container and was burned in an oxygen atmosphere.

Under nitro­gen atmosphere, 7-(di­ethyl­amino)-2-oxo-2*H*-chromene-3-carb­oxy­lic acid (298 mg, 1.14 mmol) and potassium carbonate (324 mg, 2.34 mmol) were suspended in dry *N*,*N*-di­methyl­formamide (20 ml). Allyl bromide (320 µl, 3.70 mmol) was added and the solution was stirred for 21.5 h at room temperature. After addition of water (50 ml), the mixture was extracted with di­chloro­methane (4 × 20 ml). The combined organic layer was washed with 1*M* NaOH solution (30 ml) and dried with magnesium sulfate. After filtration, the solvent was removed *in vacuo*. The crude product was purified by flash column chromatography on silica gel [di­chloro­methane:ethyl acetate = 100:0 → 80:20, *R*_f_ (di­chloro­methane:ethyl acetate = 8:2) = 0.67] to yield the title compound (256 mg, 850 µmol, 75%) as a yellow solid. A small amount of the title compound was recrystallized from acetone, leading to crystals suitable for single crystal X-ray diffraction.

Melting point: 361 K. ^1^H NMR (500 MHz, DMSO-*d*_6_, 298 K, TMS): δ = 8.59 (*s*, 1 H, *H*-4), 7.65 (*d*, *^3^J* = 9.0 Hz, 1 H, *H*-5), 6.78 (*dd*, *^3^J* = 9.0 Hz, *^4^J* = 2.5 Hz, 1 H, *H*-6), 6.54 (*d*, *^4^J* = 2.3 Hz, 1 H, *H*-8), 6.01 (*ddt*, *^2^J* = 17.2, 10.5 Hz, *^3^J* = 5.2 Hz, 1 H, C*H*=CH_2_), 5.48–5.22 (*m*, 2 H, CH=C*H*_2_), 4.72 (*dt*, *^3^J* = 5.2 Hz, *^4^J* = 1.5 Hz, 2 H, OC*H*_2_), 3.48 (*q*, *^3^J* = 7.0 Hz, 4 H, NC*H*_2_), 1.14 (*t*, *^3^J* = 7.0 Hz, 6 H, NCH_2_C*H*_3_) ppm. ^13^C NMR (125 MHz, DMSO-*d*_6_, 298 K, TMS): δ = 163.1 (*s*, *C*OOCH_2_), 158.1 (*s*, *C*-8a), 157.0 (*s*, *C*-2), 152.9 (*s*, *C*-7), 149.5 (*d*, *C*-4), 132.7 (*d*, *C*H=CH_2_), 131.9 (*d*, *C*-5), 117.6 (*t*, CH=*C*H_2_), 109.8 (*d*, *C*-6), 107.0 (*s*, *C*-4a), 106.9 (*s*, *C*-3), 95.8 (*d*, *C*-8), 64.7 (*t*, O*C*H_2_), 44.4 (*t*, N*C*H_2_), 12.3 (q, NCH_2_ *C*H_3_) ppm. MS (EI, 70 eV): *m*/*z* (%) = 301.13 (43) [*M*]^+.^, 244.10 (20) [*M* –OCH_2_CH=CH_2_]^+^. HR–MS (EI, 70 eV): found: *m*/*z* = 301.1313 [*M*]^+.^, calculated: *m*/*z* = 301.1314 [*M*]^+.^ (Δ = 0.32 ppm). IR (ATR) wavenumbers: 2972 (*w*, C—H), 1729, 1685 (*s*, C=O), 1585 (*s*, arom.), 1216, 1185, 1114 (*s*, C—O) cm^−1^. Elemental analysis C_17_H_19_NO_4_ calculated: C: 67.76, H: 6.36, N: 4.65; found: C: 67.67, H: 6.38, N: 4.54.

## Refinement   

Crystal data, data collection and structure refinement details are summarized in Table 2 ▸. The C—H hydrogen atoms were located in difference maps but were positioned with idealized geometry (methyl H atoms allowed to rotate but not to tip) and refined isotropically with *U*_iso_(H) = 1.2*U*_eq_(C) (1.5 for methyl H atoms) using a riding model.

## Supplementary Material

Crystal structure: contains datablock(s) I. DOI: 10.1107/S2056989021002218/wm5600sup1.cif

Structure factors: contains datablock(s) I. DOI: 10.1107/S2056989021002218/wm5600Isup2.hkl

Figure S1: 1H NMR spectrum of 7-(diethylamino)-2-oxo-2H-chromene-3-carboxylic acid allyl ester in dimethylsulfoxide-d6. DOI: 10.1107/S2056989021002218/wm5600sup3.tif

Figure S2: 13C NMR spectrum of 7-(diethylamino)-2-oxo-2H-chromene-3-carboxylic acid allyl ester in dimethylsulfoxide-d6. DOI: 10.1107/S2056989021002218/wm5600sup4.tif

Figure S3: Experimental (top) and calculated XRPD pattern (botom) of the title compound measured with copper radiation. DOI: 10.1107/S2056989021002218/wm5600sup5.tif

Supporting information file. DOI: 10.1107/S2056989021002218/wm5600Isup6.cml

CCDC reference: 2064943

Additional supporting information:  crystallographic information; 3D view; checkCIF report

## Figures and Tables

**Table 1:** Hydrogen-bond geometry (Å, °)

*D*—H⋯*A*	*D*—H	H⋯*A*	*D*⋯*A*	*D*—H⋯*A*
C6—H6⋯O2^i^	0.95	2.45	3.3958 (12)	171

Symmetry code: (i) -x+1, y-{\script{1\over 2}}, -z+{\script{3\over 2}}.

**Table 2:** Experimental details

Crystal data	
Chemical formula	C_17_H_19_NO_4_
*M*_r_	301.33
Crystal system, space group	Monoclinic, *P*2_1_/*c*
Temperature (K)	100
*a*, *b*, *c* (Å)	13.72487 (9), 13.05333 (9), 8.55970 (6)
β (°)	95.5220 (6)
*V* (Å^3^)	1526.40 (2)
*Z*	4
Radiation type	Cu *K*α
μ (mm^−1^)	0.77
Crystal size (mm)	0.08 × 0.06 × 0.05

Data collection	
Diffractometer	XtaLAB Synergy, Dualflex, HyPix
Absorption correction	Multi-scan (*CrysAlis PRO*; Rigaku OD, 2020 ▸)
*T*_min_, *T*_max_	0.796, 1.000
No. of measured, independent and observed [*I* > 2σ(*I*)] reflections	26198, 3125, 2975
*R*_int_	0.025
(sin θ/λ)_max_ (Å^−1^)	0.625

Refinement	
*R*[*F* ^2^ > 2σ(*F* ^2^)], *wR*(*F* ^2^), *S*	0.033, 0.090, 1.03
No. of reflections	3125
No. of parameters	202
H-atom treatment	H-atom parameters constrained
Δρ_max_, Δρ_min_ (e Å^−3^)	0.27, −0.18

Computer programs: *CrysAlis PRO* (Rigaku OD, 2020 ▸), *SHELXT* (Sheldrick, 2015*a* ▸), *SHELXL* (Sheldrick, 2015*b* ▸), *OLEX2* (Dolomanov *et al.*, 2009 ▸), *DIAMOND* (Brandenburg, 2014 ▸) and *publCIF* (Westrip, 2010 ▸).

## supplementary crystallographic information

### Crystal data

**Table:**

C_17_H_19_NO_4_	*F*(000) = 640
*M**_r_* = 301.33	*D*_x_ = 1.311 Mg m^−^^3^
Monoclinic, *P*2_1_/*c*	Cu *K*α radiation, λ = 1.54184 Å
*a* = 13.72487 (9) Å	Cell parameters from 18730 reflections
*b* = 13.05333 (9) Å	θ = 3.2–79.5°
*c* = 8.55970 (6) Å	µ = 0.77 mm^−^^1^
β = 95.5220 (6)°	*T* = 100 K
*V* = 1526.40 (2) Å^3^	Block, colorless
*Z* = 4	0.08 × 0.06 × 0.05 mm

### Data collection

**Table:**

XtaLAB Synergy, Dualflex, HyPix diffractometer	3125 independent reflections
Radiation source: micro-focus sealed X-ray tube, PhotonJet (Cu) X-ray Source	2975 reflections with *I* > 2σ(*I*)
Mirror monochromator	*R*_int_ = 0.025
Detector resolution: 10.0000 pixels mm^-1^	θ_max_ = 74.5°, θ_min_ = 3.2°
ω scans	*h* = −17→17
Absorption correction: multi-scan (CrysAlisPro; Rigaku OD, 2020)	*k* = −16→16
*T*_min_ = 0.796, *T*_max_ = 1.000	*l* = −10→9
26198 measured reflections	

### Refinement

**Table:**

Refinement on *F*^2^	Hydrogen site location: inferred from neighbouring sites
Least-squares matrix: full	H-atom parameters constrained
*R*[*F*^2^ > 2σ(*F*^2^)] = 0.033	*w* = 1/[σ^2^(*F*_o_^2^) + (0.0452*P*)^2^ + 0.4857*P*] where *P* = (*F*_o_^2^ + 2*F*_c_^2^)/3
*wR*(*F*^2^) = 0.090	(Δ/σ)_max_ < 0.001
*S* = 1.03	Δρ_max_ = 0.27 e Å^−^^3^
3125 reflections	Δρ_min_ = −0.18 e Å^−^^3^
202 parameters	Extinction correction: SHELXL (Sheldrick, 2015b), Fc^*^=kFc[1+0.001xFc^2^λ^3^/sin(2θ)]^-1/4^
0 restraints	Extinction coefficient: 0.00051 (13)

### Special details

**Table:**

Geometry. All esds (except the esd in the dihedral angle between two l.s. planes) are estimated using the full covariance matrix. The cell esds are taken into account individually in the estimation of esds in distances, angles and torsion angles; correlations between esds in cell parameters are only used when they are defined by crystal symmetry. An approximate (isotropic) treatment of cell esds is used for estimating esds involving l.s. planes.

### Fractional atomic coordinates and isotropic or equivalent isotropic displacement parameters (Å^2^)

**Table:**

	*x*	*y*	*z*	*U*_iso_*/*U*_eq_	
O1	0.42522 (5)	0.43406 (5)	0.62496 (8)	0.02005 (16)	
O2	0.30849 (5)	0.49523 (5)	0.45723 (9)	0.02685 (18)	
O3	0.14546 (5)	0.22199 (6)	0.48871 (10)	0.03164 (19)	
O4	0.15018 (5)	0.37908 (6)	0.38321 (9)	0.02571 (18)	
N1	0.69931 (6)	0.32538 (6)	0.96569 (9)	0.02028 (19)	
C1	0.33583 (7)	0.42197 (7)	0.53540 (11)	0.0200 (2)	
C2	0.28755 (7)	0.32340 (7)	0.54870 (11)	0.0200 (2)	
C3	0.33465 (7)	0.24639 (7)	0.63486 (11)	0.0208 (2)	
H3	0.302995	0.181966	0.640621	0.025*	
C4	0.42814 (7)	0.25947 (7)	0.71496 (11)	0.0196 (2)	
C5	0.48286 (7)	0.18288 (7)	0.80087 (11)	0.0213 (2)	
H5	0.457233	0.115320	0.802308	0.026*	
C6	0.57144 (7)	0.20301 (7)	0.88188 (11)	0.0208 (2)	
H6	0.606652	0.149382	0.937029	0.025*	
C7	0.61162 (7)	0.30412 (7)	0.88437 (11)	0.0187 (2)	
C8	0.55922 (7)	0.38049 (7)	0.79458 (11)	0.0194 (2)	
H8	0.584776	0.448004	0.791190	0.023*	
C9	0.47128 (7)	0.35691 (7)	0.71221 (11)	0.0184 (2)	
C10	0.18826 (7)	0.30202 (8)	0.47158 (11)	0.0220 (2)	
C11	0.05028 (7)	0.36342 (9)	0.31361 (13)	0.0278 (2)	
H11A	0.005740	0.353447	0.396689	0.033*	
H11B	0.046669	0.302041	0.245361	0.033*	
C12	0.02189 (8)	0.45623 (9)	0.22007 (14)	0.0333 (3)	
H12	0.056563	0.471426	0.132052	0.040*	
C13	−0.04888 (9)	0.51877 (10)	0.25251 (17)	0.0408 (3)	
H13A	−0.084753	0.505412	0.339854	0.049*	
H13B	−0.064001	0.577191	0.188542	0.049*	
C14	0.74215 (7)	0.42822 (8)	0.96559 (12)	0.0237 (2)	
H14A	0.730319	0.457818	0.858963	0.028*	
H14B	0.813825	0.423083	0.991818	0.028*	
C15	0.69959 (9)	0.49949 (8)	1.08247 (13)	0.0303 (2)	
H15A	0.630056	0.511206	1.049963	0.045*	
H15B	0.734605	0.564996	1.085692	0.045*	
H15C	0.706714	0.468117	1.186964	0.045*	
C16	0.74742 (7)	0.25546 (8)	1.08276 (11)	0.0231 (2)	
H16A	0.699758	0.202771	1.109081	0.028*	
H16B	0.767645	0.294469	1.179671	0.028*	
C17	0.83648 (8)	0.20262 (9)	1.02778 (14)	0.0324 (3)	
H17A	0.816019	0.157826	0.938740	0.049*	
H17B	0.868653	0.161646	1.113786	0.049*	
H17C	0.882291	0.254245	0.995308	0.049*	

### Atomic displacement parameters (Å^2^)

**Table:**

	*U*^11^	*U*^22^	*U*^33^	*U*^12^	*U*^13^	*U*^23^
O1	0.0191 (3)	0.0158 (3)	0.0244 (3)	−0.0008 (2)	−0.0025 (3)	0.0018 (3)
O2	0.0244 (4)	0.0199 (4)	0.0343 (4)	−0.0021 (3)	−0.0069 (3)	0.0057 (3)
O3	0.0251 (4)	0.0236 (4)	0.0446 (5)	−0.0067 (3)	−0.0054 (3)	0.0041 (3)
O4	0.0182 (3)	0.0256 (4)	0.0319 (4)	−0.0036 (3)	−0.0048 (3)	0.0050 (3)
N1	0.0187 (4)	0.0214 (4)	0.0205 (4)	0.0016 (3)	0.0006 (3)	0.0009 (3)
C1	0.0184 (4)	0.0192 (5)	0.0222 (5)	0.0002 (4)	−0.0001 (4)	−0.0009 (4)
C2	0.0193 (5)	0.0189 (5)	0.0218 (5)	−0.0014 (4)	0.0015 (4)	−0.0017 (4)
C3	0.0227 (5)	0.0172 (4)	0.0228 (5)	−0.0026 (4)	0.0035 (4)	−0.0015 (4)
C4	0.0212 (5)	0.0176 (5)	0.0201 (4)	−0.0004 (4)	0.0027 (4)	−0.0006 (3)
C5	0.0253 (5)	0.0163 (4)	0.0225 (5)	−0.0007 (4)	0.0030 (4)	0.0004 (4)
C6	0.0240 (5)	0.0180 (5)	0.0206 (4)	0.0036 (4)	0.0029 (4)	0.0016 (3)
C7	0.0182 (4)	0.0209 (5)	0.0173 (4)	0.0018 (4)	0.0037 (3)	−0.0010 (3)
C8	0.0200 (5)	0.0168 (4)	0.0215 (5)	−0.0008 (3)	0.0021 (4)	0.0000 (3)
C9	0.0201 (4)	0.0167 (4)	0.0188 (4)	0.0020 (3)	0.0031 (3)	0.0006 (3)
C10	0.0211 (5)	0.0201 (5)	0.0245 (5)	−0.0013 (4)	0.0013 (4)	−0.0019 (4)
C11	0.0177 (5)	0.0297 (5)	0.0348 (6)	−0.0043 (4)	−0.0044 (4)	0.0022 (4)
C12	0.0241 (5)	0.0370 (6)	0.0366 (6)	−0.0075 (5)	−0.0082 (4)	0.0101 (5)
C13	0.0346 (6)	0.0311 (6)	0.0529 (8)	−0.0015 (5)	−0.0156 (6)	0.0041 (5)
C14	0.0202 (5)	0.0249 (5)	0.0253 (5)	−0.0022 (4)	−0.0011 (4)	0.0016 (4)
C15	0.0335 (6)	0.0246 (5)	0.0317 (6)	0.0004 (4)	−0.0021 (4)	−0.0030 (4)
C16	0.0223 (5)	0.0268 (5)	0.0196 (4)	0.0027 (4)	−0.0007 (4)	0.0022 (4)
C17	0.0257 (5)	0.0366 (6)	0.0348 (6)	0.0104 (5)	0.0020 (4)	0.0064 (5)

### Geometric parameters (Å, º)

**Table:**

O1—C1	1.3916 (11)	C8—H8	0.9500
O1—C9	1.3714 (11)	C8—C9	1.3729 (13)
O2—C1	1.2062 (12)	C11—H11A	0.9900
O3—C10	1.2142 (13)	C11—H11B	0.9900
O4—C10	1.3346 (12)	C11—C12	1.4833 (15)
O4—C11	1.4558 (11)	C12—H12	0.9500
N1—C7	1.3597 (12)	C12—C13	1.3188 (19)
N1—C14	1.4655 (13)	C13—H13A	0.9500
N1—C16	1.4648 (12)	C13—H13B	0.9500
C1—C2	1.4567 (13)	C14—H14A	0.9900
C2—C3	1.3713 (14)	C14—H14B	0.9900
C2—C10	1.4824 (13)	C14—C15	1.5233 (15)
C3—H3	0.9500	C15—H15A	0.9800
C3—C4	1.4059 (13)	C15—H15B	0.9800
C4—C5	1.4134 (13)	C15—H15C	0.9800
C4—C9	1.4041 (13)	C16—H16A	0.9900
C5—H5	0.9500	C16—H16B	0.9900
C5—C6	1.3660 (14)	C16—C17	1.5173 (14)
C6—H6	0.9500	C17—H17A	0.9800
C6—C7	1.4297 (14)	C17—H17B	0.9800
C7—C8	1.4125 (13)	C17—H17C	0.9800
			
C9—O1—C1	123.53 (8)	O4—C11—H11B	110.3
C10—O4—C11	115.36 (8)	O4—C11—C12	107.12 (8)
C7—N1—C14	121.47 (8)	H11A—C11—H11B	108.5
C7—N1—C16	122.79 (8)	C12—C11—H11A	110.3
C16—N1—C14	114.62 (8)	C12—C11—H11B	110.3
O1—C1—C2	116.15 (8)	C11—C12—H12	118.2
O2—C1—O1	115.24 (8)	C13—C12—C11	123.54 (12)
O2—C1—C2	128.61 (9)	C13—C12—H12	118.2
C1—C2—C10	122.49 (9)	C12—C13—H13A	120.0
C3—C2—C1	119.67 (9)	C12—C13—H13B	120.0
C3—C2—C10	117.84 (9)	H13A—C13—H13B	120.0
C2—C3—H3	118.9	N1—C14—H14A	109.1
C2—C3—C4	122.29 (9)	N1—C14—H14B	109.1
C4—C3—H3	118.9	N1—C14—C15	112.32 (8)
C3—C4—C5	125.60 (9)	H14A—C14—H14B	107.9
C9—C4—C3	117.93 (9)	C15—C14—H14A	109.1
C9—C4—C5	116.46 (9)	C15—C14—H14B	109.1
C4—C5—H5	119.0	C14—C15—H15A	109.5
C6—C5—C4	122.04 (9)	C14—C15—H15B	109.5
C6—C5—H5	119.0	C14—C15—H15C	109.5
C5—C6—H6	119.7	H15A—C15—H15B	109.5
C5—C6—C7	120.55 (9)	H15A—C15—H15C	109.5
C7—C6—H6	119.7	H15B—C15—H15C	109.5
N1—C7—C6	121.14 (9)	N1—C16—H16A	108.9
N1—C7—C8	120.91 (9)	N1—C16—H16B	108.9
C8—C7—C6	117.90 (9)	N1—C16—C17	113.25 (8)
C7—C8—H8	120.1	H16A—C16—H16B	107.7
C9—C8—C7	119.84 (9)	C17—C16—H16A	108.9
C9—C8—H8	120.1	C17—C16—H16B	108.9
O1—C9—C4	120.09 (8)	C16—C17—H17A	109.5
O1—C9—C8	116.83 (8)	C16—C17—H17B	109.5
C8—C9—C4	123.08 (9)	C16—C17—H17C	109.5
O3—C10—O4	123.31 (9)	H17A—C17—H17B	109.5
O3—C10—C2	122.88 (9)	H17A—C17—H17C	109.5
O4—C10—C2	113.80 (8)	H17B—C17—H17C	109.5
O4—C11—H11A	110.3		
			
O1—C1—C2—C3	−5.62 (13)	C5—C6—C7—N1	179.41 (8)
O1—C1—C2—C10	174.60 (8)	C5—C6—C7—C8	−3.06 (14)
O2—C1—C2—C3	174.72 (10)	C6—C7—C8—C9	1.82 (13)
O2—C1—C2—C10	−5.06 (16)	C7—N1—C14—C15	81.70 (11)
O4—C11—C12—C13	−115.91 (12)	C7—N1—C16—C17	107.68 (11)
N1—C7—C8—C9	179.35 (8)	C7—C8—C9—O1	−178.72 (8)
C1—O1—C9—C4	−0.28 (13)	C7—C8—C9—C4	1.57 (14)
C1—O1—C9—C8	180.00 (8)	C9—O1—C1—O2	−175.17 (8)
C1—C2—C3—C4	1.44 (14)	C9—O1—C1—C2	5.12 (13)
C1—C2—C10—O3	−176.05 (10)	C9—C4—C5—C6	2.31 (14)
C1—C2—C10—O4	3.33 (13)	C10—O4—C11—C12	−179.62 (9)
C2—C3—C4—C5	−177.39 (9)	C10—C2—C3—C4	−178.77 (9)
C2—C3—C4—C9	3.51 (14)	C11—O4—C10—O3	3.12 (14)
C3—C2—C10—O3	4.17 (15)	C11—O4—C10—C2	−176.26 (8)
C3—C2—C10—O4	−176.45 (8)	C14—N1—C7—C6	178.43 (8)
C3—C4—C5—C6	−176.79 (9)	C14—N1—C7—C8	0.97 (13)
C3—C4—C9—O1	−4.15 (13)	C14—N1—C16—C17	−84.26 (11)
C3—C4—C9—C8	175.56 (9)	C16—N1—C7—C6	−14.31 (13)
C4—C5—C6—C7	0.96 (14)	C16—N1—C7—C8	168.23 (8)
C5—C4—C9—O1	176.67 (8)	C16—N1—C14—C15	−86.53 (10)
C5—C4—C9—C8	−3.62 (14)		

### Hydrogen-bond geometry (Å, º)

**Table:**

*D*—H···*A*	*D*—H	H···*A*	*D*···*A*	*D*—H···*A*
C6—H6···O2^i^	0.95	2.45	3.3958 (12)	171

Symmetry code: (i) −*x*+1, *y*−1/2, −*z*+3/2.
